# Mechanochemical Lignin-Mediated Strecker Reaction

**DOI:** 10.3390/molecules22010146

**Published:** 2017-01-17

**Authors:** Saumya Dabral, Mathias Turberg, Andrea Wanninger, Carsten Bolm, José G. Hernández

**Affiliations:** 1 Institute of Organic Chemistry, RWTH Aachen University, Landoltweg 1, D-52074 Aachen, Germany; saumya.dabral@oc.rwth-aachen.de (S.D.); mathias.turberg@rwth-aachen.de (M.T.); 2 Angewandte Organische Chemie, Hochschule Niederrhein, University of Applied Sciences, Adlerstr. 32, D-47798 Krefeld, Germany; andrea.wanninger@hs-niederrhein.de

**Keywords:** mechanochemistry, lignin, Strecker reaction, multicomponent reactions, ball milling

## Abstract

A mechanochemical Strecker reaction involving a wide range of aldehydes (aromatic, heteroaromatic and aliphatic), amines, and KCN afforded a library of α-aminonitriles upon mechanical activation. This multicomponent process was efficiently activated by lignocellulosic biomass as additives. Particularly, commercially available Kraft lignin was found to be the best activator for the addition of cyanide to the in situ formed imines. A comparative study of the ^31^P-NMR (Nuclear Magnetic Resonance) along with IR (Infrared) data analysis for the Kraft lignin and methylated Kraft lignin samples ascertained the importance of the free hydroxyl groups in the activation of the mechanochemical reaction. The solvent-free mechanochemical Strecker reaction was then coupled with a lactamization process leading to the formation of the *N*-benzylphthalimide (**5a**) and the isoindolinone derivative **6a**.

## 1. Introduction

Mechanochemistry—the use of mechanical energy to induce chemical transformation—has flourished in recent years, providing chemists with the means to improve synthetic chemical design in organic, inorganic, organometallic, and supramolecular chemistry, among others [1,2,3,4,5]. Commonly, mechanochemical reactions are conducted by milling, grinding, shearing, or pulling reactants in the absence of organic solvents or in the presence of a catalytic amount of them (liquid-assisted grinding, LAG) [6]. In organic chemistry, the use of electric ball mills—which until very recently had been used mostly to reduce the particle size of materials (rocks, inorganics, cellulose, lignin, etc.)—has enabled the study of numerous metal-catalyzed transformations [7], organocatalytic [8] and enzymatic processes [9], lignin [10] and cellulose depolymerizations [11,12,13,14], multicomponent reactions [15], and many others [16,17,18,19]. As a result of these investigations, mechanochemical activation and procedures have revealed several benefits, such as short reaction milling times, higher yield, reduced waste production, improved selectivity, and stoichiometry control, among others.

**Figure 1:**
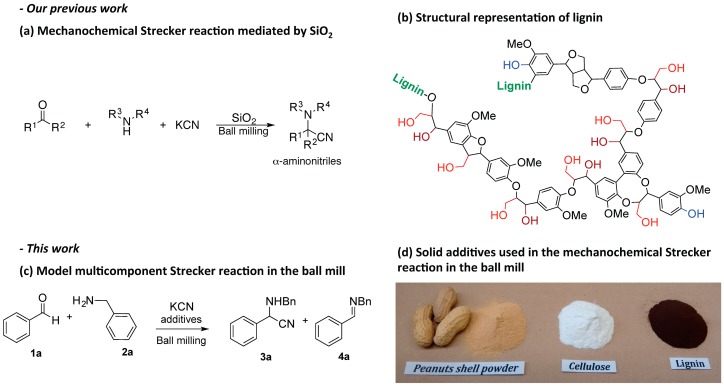
(**a**) Mechanosynthesis of α-aminonitriles mediated by SiO_2_; (**b**) Structure of lignin; (**c**) General mechanochemical Strecker reaction; (**d**) Natural heterogeneous additives tested as activators for the mechanochemical Strecker reaction.

In the field of multicomponent reactions, our group very recently reported the use of inexpensive and readily available silica gel to mediate the mechanochemical multicomponent Strecker reaction (Figure 1a) [20]. The efficiency of this protocol enabled the high-yielding mechanosynthesis of diverse α-aminonitriles and some tetrahydroisoquinoline derivatives.

In the search for alternative materials capable of mediating the Strecker reaction under ball milling conditions, we focused our attention on the utilization of the main constituents of lignocellulosic feedstocks—such as cellulose or lignin—as potential activators for the reaction between aldehydes (or ketones), amines, and potassium cyanide in the ball mill. Cellulose is a readily available homopolymer of glucose consisting of β(1–4) bonds, which has been recently used as an additive and as a chiral inducer in organic reactions [21]. On the other hand, lignin is an inexpensive and abundant biopolymer containing phenylpropanoid units that are linked together through a variety of C-C and C-O bonds, resulting in the presence of aliphatic hydroxyl and phenolic hydroxyl groups (Figure 1b) [22]. The structural features present in cellulose and lignin are envisioned to confer these additives Brønsted acidic functionality, which could be useful for the activation of organic reactions [23]. Therefore, in order to evaluate the ability of natural heterogeneous activators to perform under mechanochemical conditions, we decided to test the catalytic activity of peanut shell powder (PSP) as a raw source of lignocellulosic biomass, cellulose, and several lignin samples in the three-component Strecker reaction to afford α-aminonitriles (Figure 1c–d).

## 2. Results and Discussion

### 2.1. Lignocellulosic Biomass as Activator of the Mechanochemical Strecker Reaction

**Figure 2:**
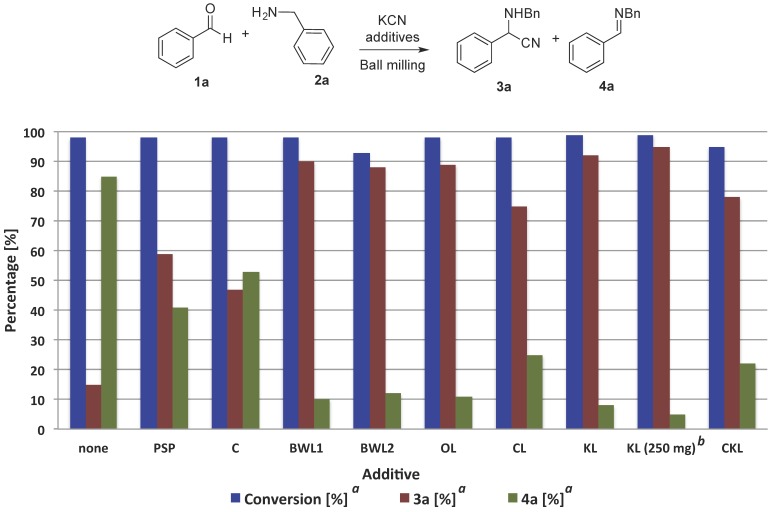
Conversion and selectivity of the mechanochemical Strecker reaction using biomass-derived additives. Reaction conditions: unless otherwise stated, **1a** (53.1 mg, 0.5 mmol), **2a** (53.6 mg, 0.5 mmol), KCN (35.8 mg, 0.55 mmol), and the additive (200 mg) were milled in a mixer mill for 2 h at 30 Hz using a stainless steel milling jar of 10 mL and one milling ball of the same material 10 mm in diameter. ^a^ Determined by ^1^H-NMR spectroscopy; ^b^ 3 h of milling. PSP = peanut shell powder; C = cellulose; BWL1 = beechwood lignin 1; BWL2 = beechwood lignin 2; OL = oak lignin; CL = cherry lignin; KL = Kraft lignin; CKL = capped Kraft lignin.

In order to evaluate the effect of the aforementioned solid additives on the Strecker reaction, we began ball milling a mixture of benzaldehyde (**1a**), benzylamine (**2a**), and potassium cyanide in a mixer ball mill. The analysis of the reaction mixture by ^1^H-NMR spectroscopy after two hours of milling at 30 Hz indicated full consumption of **1a** and **2a**. Additionally, the ^1^H-NMR analysis also revealed the presence of the expected α-aminonitrile **3a** in minor quantity, along with the aldimine **4a** as the major product preceding the milling process (conversion 98%, **3a**/**4a**; 15/85; Figure 2). The formation of the α-aminonitrile **3a** in 15% upon milling is remarkable, since the same uncatalyzed reaction attempted in solution (acetonitrile) afforded mainly imine **4a** and traces of the product **3a**. Next, the reaction was repeated in the presence of peanut shell powder (PSP) as an additive for the multicomponent reaction. To our delight, under these conditions, the amount of the α-aminonitrile **3a** rose significantly after milling (**3a/4a**; 59/41; Figure 2). With the aim of examining the role of some of the major components present in PSP (cellulose, hemicellulose, lignin), crystalline cellulose and beechwood lignin (BWL1) were tested under mechanochemical conditions. After 2 h of milling, in comparison to cellulose, the lignin sample proved to be highly active in promoting the Strecker reaction, which resulted in a mixture of α-aminonitrile:imine of 90:10 (Figure 2).

Subsequently, different lignin samples (cherry, beechwood, and Kraft) were examined as additives in the mechanochemical multicomponent reaction. In general, all the lignin samples clearly exhibited a positive effect favoring the formation of the product **3a** over the imine **4a** (Figure 2). Among the activators tested, commercial Kraft lignin showed the highest catalytic activity (**3a**/**4a**; 92/8; Figure 2) after 2 h at 30 Hz. An increase in the lignin loading (from 200 mg to 250 mg) and time (from 2 h to 3 h) led to higher **3a**:**4a** ratio of 95:5. However, higher additive loading or longer milling times had a negligible impact on the output of the mechanochemical reaction. Finally, control experiments under conventional stirring in organic solvents (CD_3_CN and CDCl_3_) in the presence of Kraft lignin (KL) revealed incomplete conversion of starting materials **1a** and **2a**. Furthermore, the selectivity of the reaction in solution was found to mostly favor the formation of the imine **4a** (in CD_3_CN: **3a**/**4a**; 47/53 and in CDCl_3_: **3a**/**4a**; 70/30).

### 2.2. Investigation of the Role of the Kraft Lignin in the Mechanochemical Reaction

**Figure 3:**
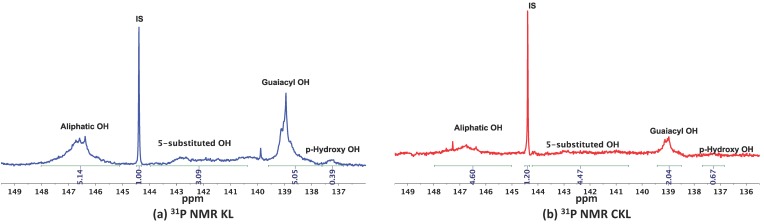
Functional group analysis of lignin by quantitative ^31^P-NMR measurements after phosphitylation; (**a**) Kraft Lignin (KL); (**b**) capped Kraft lignin (CKL) with dimethyl carbonate (DMC). IS = internal standard.

To determine the role of the Brønsted acidic sites of lignin on the activation of the multicomponent reaction, a sample of Kraft lignin was capped (methylated) following the procedure reported by Argyropoulos and coworkers using dimethyl carbonate (DMC) in the presence of NaOH in DMSO [24]. Under these conditions, the hydroxyl groups present in lignin are known to undergo methylation, which could have a direct impact on the overall functionality of the lignin, hence affecting its reactivity. Indeed, the mechanochemical reaction of **1a**, **2a**, and KCN in the presence of capped Kraft lignin (CKL) resulted in a slight decrease in the conversion of the reactants, and in lowering the amount of expected α-aminonitrile **3a** (conv. 95%, **3a**/**4a**; 78/22; Figure 2). Analysis by ^31^P-NMR [25] (Figure 3 and Table 1) and IR spectroscopy (Supplementary Material, Figure S1) of the lignin samples KL and CKL marked a clear reduction in the hydroxyl group content in the CKL compared to the raw KL. As observed in Figure 3, the content of aliphatic and phenolic OH groups decreased considerably after the capping reaction with DMC, which correlates well to the lower catalytic activity observed experimentally using CKL as additive.

Finally, the recyclability of Kraft lignin was evaluated. Both filtration and centrifugation of the reaction mixture after milling allowed the expedient separation of the lignin additive. This recovered lignin sample was then reused in the standard Strecker reaction. Again, the mechanochemical milling of **1a**, **2a**, KCN, and recycled KL showed full consumption of the benzaldehyde (**1a**) and the benzylamine (**2a**), although now the ^1^H-NMR analysis revealed a decrease in selectivity of the reaction (fresh KL: **3a**/**4a**; 92/8, recycled KL: **3a**/**4a**; 62/38).

### 2.3. Scope of the Mechanochemical Strecker Reaction

With the optimized conditions, we evaluated the scope of the lignin-mediated Strecker reaction in the ball mill. Starting with benzylamine (**2a**) as amine partner, benzaldehydes substituted either with electron-donating functional groups (**1b**–**c**) or aromatic aldehydes bearing electron-withdrawing substituents were tested, giving the corresponding α-aminonitriles **3b**–**f** as the major products after three hours of milling (entries 2–6 in Table 2). Similarly, heterocyclic and aliphatic aldehydes such as 3-pyridinecarboxaldehyde (**1g**) (Supplementary Materials, Figure S2) and cyclohexanecarboxaldehyde (**1h**) also proved to be suitable for the multicomponent reaction in the ball mill, affording the products (**4g**–**h**) preferentially. Finally, a set of experiments using aniline (**2b**) as amino component with **1a** (entry 9 in Table 2), and cyclohexanone (**1i**) as carbonyl compound together with benzylamine (**2a**) (entry 10 in Table 2) were also successful, highlighting the wide range of applicability for the mechanochemical method.

**Scheme 1:**
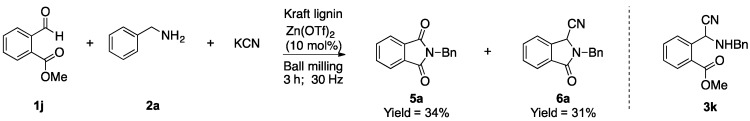
Mechanosynthesis of *N*-benzylphthalimide (**5a**) and isoindolinone (**6a**).

Next, in order to further exhibit the suitability of the mechanochemical Strecker reaction, we extended the protocol to the reaction between 2-(methoxycarbonyl)benzaldehyde (**1j**), **2a**, and KCN. The expected α-aminonitrile **3k** could in principle undergo a lactamization process in the milling vessel, leading to the formation of the isoindolinone derivative **6a** (Scheme 1) [26].

**Scheme 2:**
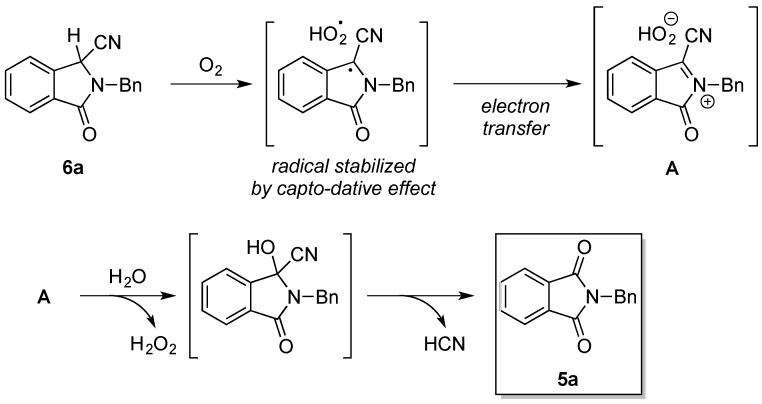
Possible reaction mechanism for the formation of **5a** in the ball mill.

In the presence of Kraft lignin, the milling process instead afforded the *N*-benzylphthalimide (**5a**) (33%). The formation of **5a** can be explained as a result of a cyanide-catalyzed oxidative amidation of the isoindolinone **6a** under the aerobic reaction conditions (ambient atmosphere) present in the ball mill (Scheme 2) [27,28,29]. Next, the same reaction was repeated, but this time Zn(OTf)_2_ (10 mol%) was added in addition to Kraft lignin in the milling vessel. Under these conditions, the mechanical milling led to the formation of the products **5a** and **6a** in 34% and 31% yield (Scheme 1). A control reaction using only Zn(OTf)_2_ (10 mol %) as a catalyst showed traces of the products **5a** and **6a**.

## 3. Materials and Methods

### 3.1. Materials

Aldehydes, ketone, amines, and KCN were purchased commercially and used without further purification. Cellulose (50 μm) was obtained from (J&K Scientific GmbH, Freiburgerstrasse 11, D-75179 Pforzheim, Germany).

Peanut shell powder was obtained after milling pieces of peanut shells (5 g) in a FRITSCH Planetary micro mill model “Pulverisette 7 classic line”, using milling vessels made of ZrO_2_ (45 mL) with five milling balls of the same material (10 mm in diameter) for 30 min at 600 rpm.

Dioxasolv cherry and oak lignins were extracted following a previously established literature procedure [30]. To cherry or oak sawdust (200 g) in a 2 L flask was added 1,4-dioxane (1.5 L) followed by the addition of 2 N HCl (0.16 L) solution. The following mixture was heated to a gentle reflux under an N_2_ atmosphere for 1 hour. The mixture was then cooled down to room temperature, and the lignin-containing liquor was collected by filtration. The collected liquor was concentrated under vacuum, resulting in a gummy residue, which was then taken up in acetone/water (9:1, 250 mL) and precipitated by the addition to rapidly stirring water (2.5 L). The crude lignin was collected by filtration and dried under vacuum. The dried crude lignin was taken up in acetone/methanol (9:1) and precipitated by dropwise addition to rapidly stirring Et_2_O (2 L). The precipitated lignin was collected by filtration and dried under vacuum to give purified samples of cherry/oak lignin (approximately 20 g). These lignins were used in subsequent experiments without further processing.

Organosolv beechwood Lignin (BWL1) was obtained after a pulping process using mixtures of ethanol and water.

Organosolv beechwood Lignin (BWL2) was extracted from beechwood chips using the following ethanol-based organosolv process. The lignin was extracted with aqueous ethanol (50% *w/w*) without the addition of an acid catalyst. Lignin was then precipitated with water and afterwards washed with water to remove the residual carbohydrates. The precipitate was sedimented by centrifugation, and the liquor above decanted. Finally, the lignin was dried and pulverized.

Kraft lignin-370959 was purchased from Sigma-Aldrich (Sigma-Aldrich Co., St. Louis, MO, USA).

### 3.2. Methods

#### 3.2.1. Monitoring of the reactions and purification of the products

Reactions were monitored by thin-layer chromatography using TLC plates with silica gel 60 on aluminum with fluorescence indicator F254 from MERCK. Qualitative analysis of the TLC plates was carried out using UV light (λ = 254 nm and λ = 366 nm) or by immersion in an aqueous solution of potassium permanganate (KMnO_4_).

Column chromatography was performed using silica gel 60 (40−63 μm) and distilled grade solvents (*n*-pentane/ethyl acetate) as eluent.

#### 3.2.2. Characterization of the Products **3**, **4**, **5** and **6** (NMR, IR Spectroscopy, MS Spectrometry)

All NMR spectra were recorded on 300, 400, and 600 MHz spectrometers (Varian Mercury 300, Varian V-NMRS 300 and 600). Proton chemical shifts are reported in parts per million on the δ scale and are calibrated using the residual non-deuterated solvent signal as an internal reference: CDCl_3_ (δ 7.26 ppm). Carbon chemical shifts are reported in parts per million on the δ scale and are referenced to the centermost carbon signal of the deuterated solvent: CDCl_3_ (δ 77.16 ppm). Spectral data is provided as follows: chemical shift in ppm (from downfield to upfield), multiplicity (s = singlet, d = doublet, dd = doublet of doublets, t = triplet, br = broad, m = multiplet), coupling constant J in Hz, and integration. IR-spectra were recorded on a Perkin Elmer 100 FT/IR spectrometer (PerkinElmer Life and Analytical Sciences, Shelton, 710 Bridgeport Avenue, CT, USA). Mass spectra were acquired on a Finnigan SSQ7000 (EI (Electron Ionization), 70 eV) spectrometer (Finningan Corporation, Waltham, MA, USA).

#### 3.2.3. Mechanochemical Synthesis

Caution! Potassium cyanide is toxic and may also release highly toxic hydrogen cyanide. All operations involving KCN should be conducted in a well-ventilated fume hood.

Typical procedure for the Strecker reaction in the mixer mill: A mixture of the carbonyl compound (1; 0.50 mmol), amine (2; 0.50 mmol), KCN (35.8 mg, 0.55 mmol), and additive was milled in a 10 mL stainless steel milling vessel with one stainless steel milling ball of 10 mm in diameter at 30 Hz for 3 h. After the milling was complete, the content of one milling vessel was transferred into a beaker using a small amount of organic solvent (DCM or ethyl acetate). Then, a known amount of the internal standard (1,3,5-trimethoxy-benzene) was added, and the mixture was filtered to remove the insoluble material. Next, the filtrate was evaporated under reduced pressure. The residue was dissolved in CDCl_3_ and analyzed by ^1^H-NMR spectroscopy to determine the yield. The content of the second milling vessel was filtered with ethyl acetate, washed with aqueous saturated NaHCO_3_ solution and brine. The combined organic layers were dried over anhydrous Na_2_SO_4_, concentrated in vacuum, and further dried under reduced pressure to give the product.

#### 3.2.4. Capping of Kraft Lignin with Dimethyl Carbonate

Kraft lignin (1 g) was dissolved in dimethyl sulfoxide (20 mL). Then, NaOH (240 mg) and DMC (6 mL) were added to the above mixture. The reaction mixture was heated at 150 °C for 5 h. After this time, the reaction mixture was cooled to room temperature, and then 2 N HCl (100 mL) was added to the mixture to precipitate the lignin. The precipitated lignin was transferred to a filter, and washed with water (3 × 100 mL). The solid residue was then dried overnight under vacuum at 50 °C.

#### 3.2.5. ^31^P-NMR Spectroscopy

The quantitative ^31^P-NMR analysis for the phenolic and the aliphatic hydroxyl groups in the lignins—both before and after the DMC capping reaction—were conducted using a standard phosphorus pulse program following previous literature reports [25]. A weighed amount of vacuum-dried lignin sample (30 mg) was dissolved in 700 µL of an anhydrous solvent mixture (pyridine:CDCl_3_ 1.6:1, *v/v*). A cyclohexanol solution (100 µL, 10.85 mg·mL^−1^) was added as an internal standard along with 100 µL of chromium (III) acetylacetonate solution (5.2 mg·mL^−1^) as the relaxation reagent. Finally, 100 µL of the phosphitylating agent (2-chloro-4,4,5,5-tetramethyl-1,3,2-dioxaphospholane) was added, and the mixture was transferred into a 5 mm-NMR tube for-NMR acquisition using 512 scans and a relaxation delay of 10 min on a 300 MHz spectrometer.

## 4. Conclusions

In summary, we first demonstrated the use of peanut shell powder followed by the cellulose and lignin components of lignocellulosic biomass as solid promoters for the mechanochemical Strecker reaction. Out of the set of lignin samples tested, commercial Kraft lignin was found to be the best activator to enhance the cyanation step of imines in the ball mill. The relationship between the molecular architecture of lignin and its catalytic activity is believed to be highly complex. In this regard, ^31^P-NMR and IR analysis of raw Kraft lignin and methylated (capped) lignin highlighted the role of the hydroxyl group content in lignin with its ability to facilitate the formation of the α-aminonitriles. Kraft lignin also proved to be very effective at promoting the multicomponent Strecker reaction between several aldehydes (aromatic, heteroaromatic, and aliphatic), amines, and KCN. Furthermore, improvement in the separation method of the lignin after the reaction—which enables higher recyclability of this additive—is currently ongoing in our group. Finally, the high efficiency of the mechanochemical approach was further demonstrated during the solvent-free synthesis of the *N*-benzylphthalimide (**5a**) and the isoindolinone (**6a**) through a one-pot three-component Strecker-lactamization catalyzed by the system Zn(OTf)_2_-Kraft lignin in the ball mill.

## Figures and Tables

**Table 1:** Hydroxyl content quantification by ^31^P-NMR spectroscopy of KL and CKL.

	OH	OH (mmol/g)				
Lignin		Aliphatic OH	5-Substituted OH	Guaiacyl OH	p-Hydroxyphenyl OH	Total Phenolic OH
KL		1.79	0.90	1.77	0.17	2.84
CKL		0.7	0.3	0.5	0.04	0.84

**Table 2:**
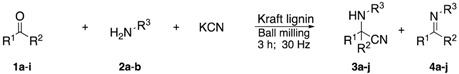
Mechanochemical Strecker reaction of aldehydes, ketones, and various amines ^a^.

Entry	Carbonyl Compound	Amine	α-aminonitrile 3a–j	Imine 4a–j	Yield [3:4] (%) ^b^
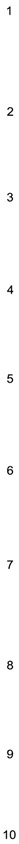	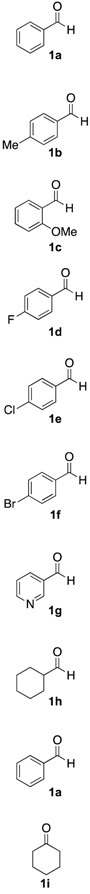	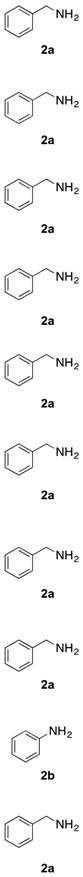	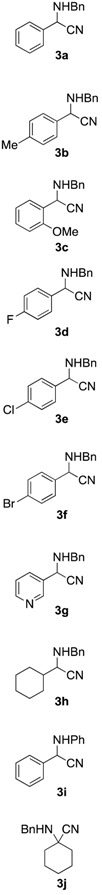	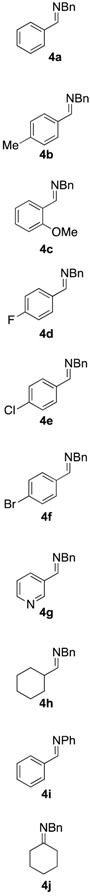	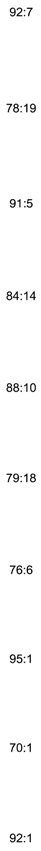

^a^ Reaction conditions: carbonyl compound (0.5 mmol), amine (0.5 mmol), KCN (35.8 mg, 0.55 mmol), and Kraft lignin (250 mg) were milled in a mixer mill at 30 Hz for 3 h using a 10 mL stainless steel milling jar with 1 ball of 10 mm in diameter of the same material; ^b^ Determined by ^1^H-NMR spectroscopy with 1,3,5-trimethoxy-benzene as the internal standard.

## Supplementary Materials

The following are available online at: http://www.mdpi.com/1420-3049/22/1/146/s1, Figure S1–S2 and characterization of the products **3a**–**j**, **5a** and **6a**.

## Abbreviations

BWL	Beachwood lignin
C	Cellulose
CKL	Capped Kraft lignin
CL	Cherry lignin
DMC	Dimethyl carbonate
IS	Internal standard
KL	Kraft lignin
LAG	Liquid assisted grinding
OL	Oak lignin
PSP	Peanut shell powder
