# Supplementary material for: Mechanochemical Lignin-Mediated Strecker Reaction
Source: Molecules. 2017 Jan 17;22(1):146. doi: 10.3390/molecules22010146 (PMC6155632; doi:10.3390/molecules22010146)
Supplement: Supplementary file 1 [file molecules-22-00146-s001.pdf]

Supplementary Materials: Mechanochemical Lignin-Mediated Strecker Reaction

Saumya Dabral, Mathias Turberg, Andrea Wanninger, Carsten Bolm and José G. Hernández

Contents

1. Figure S1. IR spectra of Kraft lignin and capped Kraft lignin 2
2. Figure S2. Monitoring of the mechanochemical Strecker reaction of **1g**, **2a**, and KCN by ^{1}H NMR spectroscopy. 3
3. Characterization of the products **3a–j**, **5a** and **6a**. 3
4. References 5

1. Figure S1

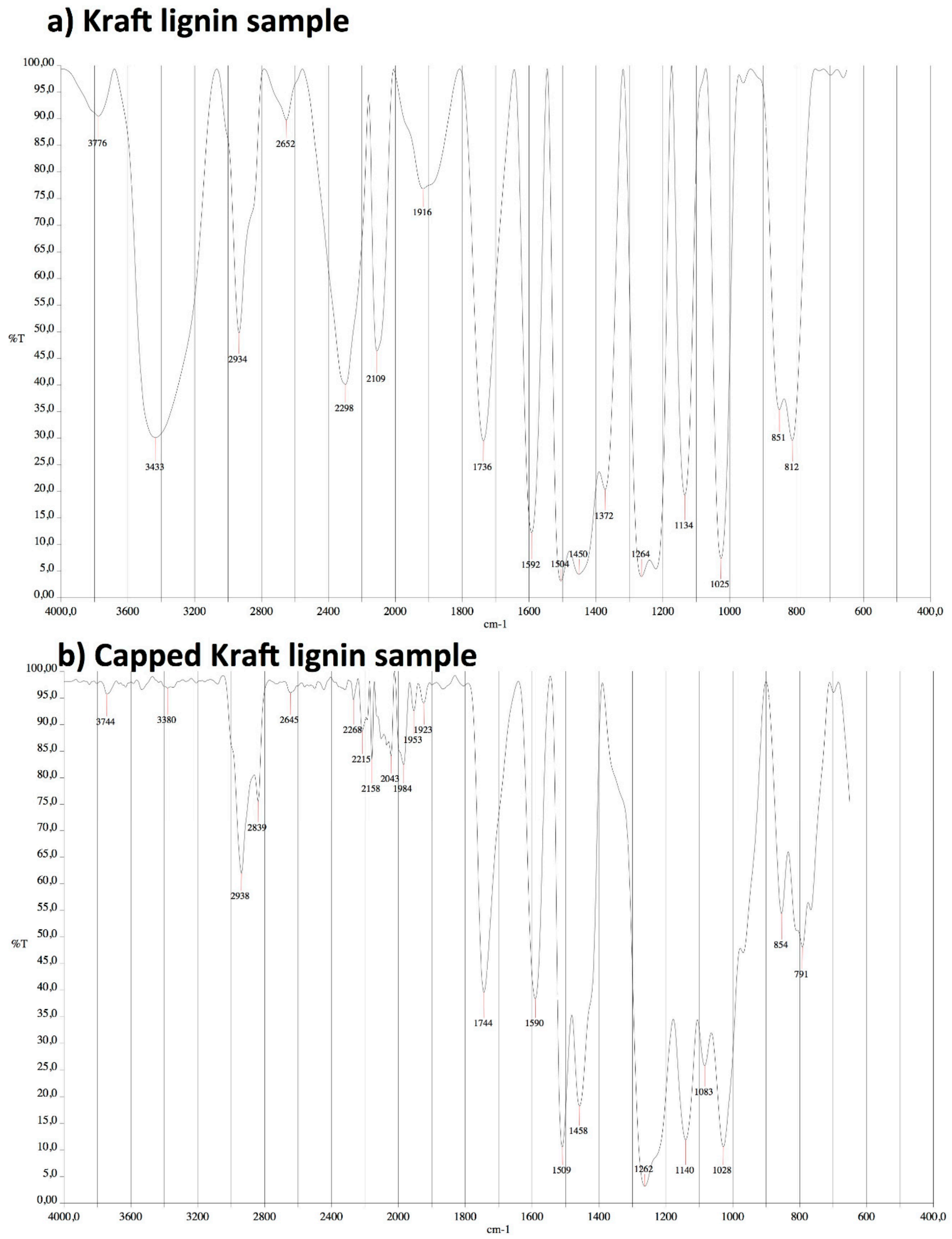

Figure S1. IR spectra of Kraft lignin and capped Kraft lignin.

2. Figure S2

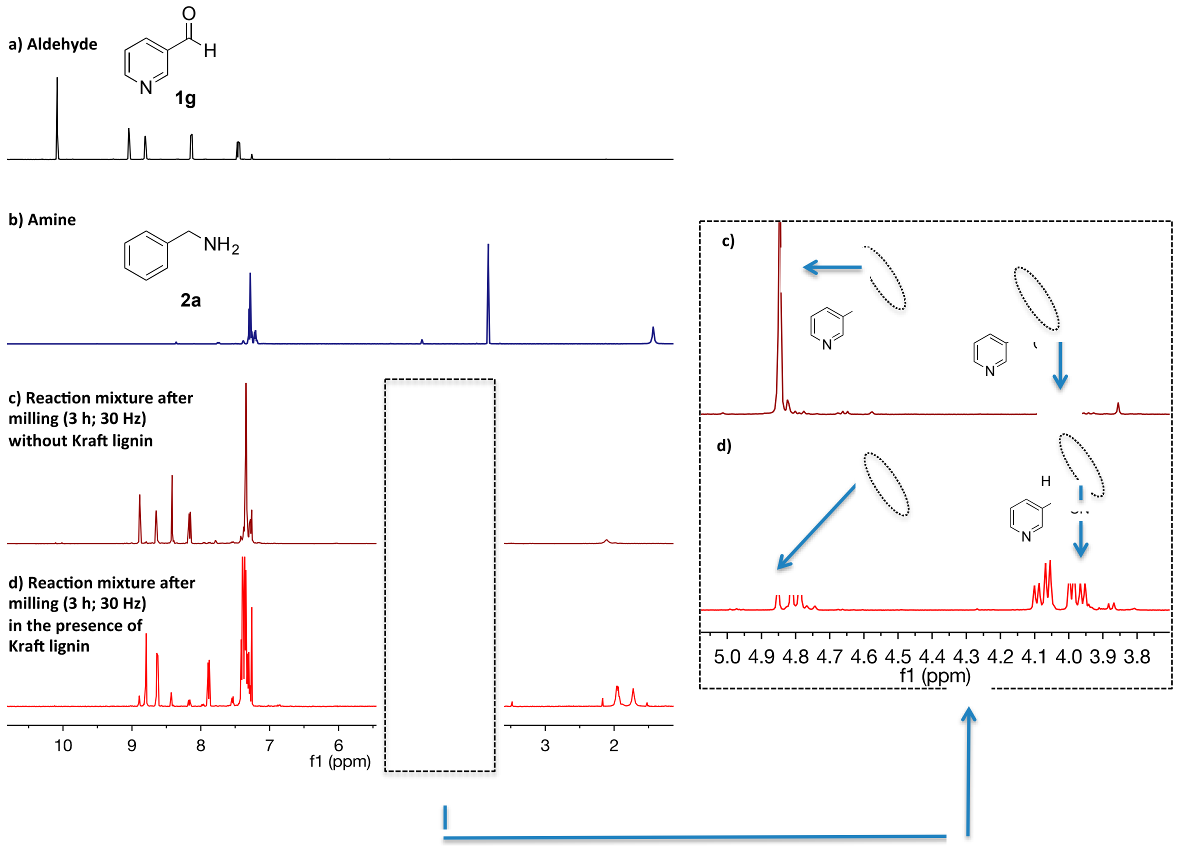

Figure S2. Monitoring of the mechanochemical Strecker reaction of **1g**, **2a**, and KCN by ^{1}H NMR spectroscopy.

3. Characterization of the Products 3a-j, 5a and 6a

2-(Benzylamino)-2-phenylacetonitrile (3a) [1,2]

^{1}H NMR(600 MHz, CDCl$_3$) δ (ppm) = 7.56 (dd, *J* = 7.9 Hz, *J* = 1.4 Hz, 2H), 7.45–7.40 (m, 4H), 7.40–7.36 (m, 3H), 7.33–7.29 (m, 1H), 4.76 (s, 1H), 4.08 (d, *J* = 13.0 Hz, 1H), 3.97 (d, *J* = 13.0 Hz, 1H), 1.89 (br s, 1H).
^{13}C NMR (150 MHz, CDCl$_3$) δ (ppm) = 138.2, 134.8, 129.1, 129.0, 128.7, 128.5, 127.7, 127.4, 118.8, 53.5, 51.3.

2-(Benzylamino)-2-(*p*-tolyl)acetonitrile (3b) [1,3]

^{1}H NMR (600 MHz, CDCl$_3$) δ (ppm) = 7.42 (dd, *J* = 7.5 Hz, *J* = 5.7 Hz, 4H), 7.37 (ddd, *J* = 7.6 Hz, *J* = 6.6 Hz, *J* = 1.4 Hz, 2H), 7.31 (ddd, *J* = 6.7 Hz, *J* = 6.3 Hz, *J* = 1.6 Hz, 1H), 7.22 (d, *J* = 7.9 Hz, 2H), 4.72 (s, 1H), 4.06 (d, *J* = 13.0 Hz, 1H), 3.95 (d, *J* = 13.1 Hz, 1H), 2.38 (s, 3H), 1.87 (br. s, 1H).
^{13}C NMR (150 MHz, CDCl$_3$) δ (ppm) = 139.1, 138.3, 131.9, 129.7, 128.8, 128.5, 127.7, 127.3, 119.0, 53.3, 51.4, 21.3.

2-(Benzylamino)-2-(2-methoxyphenyl)acetonitrile (3c) [4]

^{1}H NMR (600 MHz, CDCl$_3$) δ (ppm) = 7.41 (d, *J* = 8.0 Hz, 2H), 7.39–7.35 (m, 4H), 7.33–7.29 (m, 1H), 7.00 (td, *J* = 7.5 Hz, *J* = 1.0 Hz, 1H), 6.95 (dd, *J* = 8.1 Hz, *J* = 1.0 Hz, 1H), 4.83 (s, 1H), 4.09 (d, *J* = 13.0 Hz, 1H), 3.95 (d, *J* = 13.0 Hz, 1H), 3.88 (s, 3H), 2.30 (br s, 1H).
^{13}C NMR (150 MHz, CDCl$_3$) δ (ppm) = 156.9, 138.4, 130.6, 128.9, 128.6, 128.5, 127.6, 123.3, 121.0, 119.1, 111.3, 55.7, 51.6, 49.4.

2-(Benzylamino)-2-(4-fluorophenyl)acetonitrile (3d) [5]
^{1}H NMR (600 MHz, CDCl$_3$) δ (ppm) = 7.57–7.51 (m, 2H), 7.43–7.39 (m, 2H), 7.39–7.34 (m, 2H), 7.33–7.28 (m, 1H), 7.13–7.07 (m, 2H), 4.74 (s, 1H), 4.06 (d, *J* = 12.9 Hz, 1H), 3.95 (d, *J* = 12.9 Hz, 1H), 1.89 (br s, 1H).
^{13}C NMR (150 MHz, CDCl$_3$) δ (ppm) = 163.1 (d, C_{ipso}–F, $^1J_{CF}$ = 248.2 Hz), 138.0, 130.7 (d, C_{para}–F, $^4J_{CF}$ = 3.1 Hz), 129.3 (d, C_{meta}–F, $^3J_{CF}$ = 8.5 Hz), 128.8, 128.5, 127.8, 118.7, 116.0 (d, C_{ortho}–F, $^2J_{CF}$ = 21.6 Hz), 52.8, 51.3.

2-(Benzylamino)-2-(4-chlorophenyl)acetonitrile (3e) [6]

^{1}H NMR (400 MHz, CDCl$_3$) δ (ppm) = 7.54–7.46 (m, 2H), 7.43–7.39 (m, 2H), 7.39–7.25 (m, 5H), 4.73 (s, 1H), 4.05 (d, *J* = 13.0 Hz, 1H), 3.95 (d, *J* = 13.0 Hz, 1H), 1.89 (br s, 1H).
^{13}C NMR (100 MHz, CDCl$_3$) δ (ppm) = 138.0, 135.2, 133.3, 129.3, 128.8, 128.8, 128.5, 127.9, 118.5, 52.9, 51.3.

2-(Benzylamino)-2-(4-bromophenyl)acetonitrile (3f) [6]

^{1}H NMR (400 MHz, CDCl$_3$) δ (ppm) = 7.58–7.52 (m, 2H), 7.47–7.33 (m, 6H), 7.33–7.27 (m, 1H), 4.72 (s, 1H), 4.05 (d, *J* = 12.9 Hz, 1H), 3.95 (d, *J* = 13.0 Hz, 1H), 1.89 (br s, 1H).
^{13}C NMR (100 MHz, CDCl$_3$) δ (ppm) = 138.0, 133.9, 132.2, 129.1, 128.8, 128.5, 127.9, 123.3, 118.4, 53.0, 51.3.

2-(Benzylamino)-2-(pyridin-3-yl)acetonitrile (3g) [7,8]

^{1}H NMR (600 MHz, CDCl$_3$) δ (ppm) = 8.77 (d, *J* = 2.4 Hz, 1H), 8.60 (dd, *J* = 4.8 Hz, 1.7 Hz, 1H), 7.87 (ddd, *J* = 8.0 Hz, *J* = 2.9 Hz, *J* = 1.4 Hz, 1H), 7.41–7.37 (m, 2H), 7.37–7.32 (m, 3H), 7.31–7.27 (m, 1H), 4.78 (s, 1H), 4.05 (d, *J* = 13.0 Hz, 1H), 3.95 (d, *J* = 12.9 Hz, 1H), 2.06 (br s, 1H).
^{13}C NMR (150 MHz, CDCl$_3$) δ (ppm) = 150.4, 148.8, 137.7, 135.0, 130.7, 128.8, 128.5, 127.9, 123.7, 117.8, 51.3, 51.3.

2-(Benzylamino)-2-cyclohexylacetonitrile (3h) [8]

^{1}H NMR (400 MHz, CDCl$_3$) δ (ppm) = 7.43–7.26 (m, 5H), 4.08 (d, *J* = 13.0 Hz, 1H), 3.82 (d, *J* = 13.0 Hz, 1H), 3.31 (d, *J* = 6.3 Hz, 1H), 1.94–1.62 (m, 6H), 1.34–1.07 (m, 5H).
^{13}C NMR (100 MHz, CDCl$_3$) δ (ppm) = 138.6, 128.6, 128.4, 127.6, 119.7, 55.6, 51.9, 40.8, 29.8, 29.0, 26.1, 25.8, 25.7.

2-Phenyl-2-(phenylamino)acetonitrile (3i) [2,6]

^{1}H NMR (400 MHz, CDCl$_3$) δ (ppm) = 7.65–7.58 (m, 2H), 7.51–7.41 (m, 3H), 7.32–7.25 (m, 2H), 6.95–6.89 (m, 1H), 6.82–6.76 (m, 2H), 5.44 (s, 1H), 4.05 (br s, 1H).
^{13}C NMR (100 MHz, CDCl$_3$) δ (ppm) = 144.8, 134.1, 129.7, 129.7, 129.5, 127.4, 120.4, 118.3, 114.3, 50.4.

1-(Benzylamino)cyclohexane-1-carbonitrile (3j) [9]

^{1}H NMR (400 MHz, CDCl$_3$) δ (ppm) = 7.42–7.25 (m, 5H), 3.92 (s, 2H), 2.11–2.01 (m, 2H), 1.85–1.76 (m, 2H), 1.73–1.51 (m, 5H), 1.37–1.22 (m, 1H).
^{13}C NMR (100 MHz, CDCl$_3$) δ (ppm) = 139.5, 128.7, 128.5, 127.5, 122.3, 57.5, 48.7, 36.2, 25.3, 22.4.

***N*-Benzylphthalimide (5a)** [10]

^{1}H NMR (400 MHz, CDCl$_3$) δ (ppm) = 7.85 (dd, *J* = 5.4, 3.1 Hz, 2H), 7.70 (dd, *J* = 5.5, 3.1 Hz, 2H), 7.46-7.41 (m, 2H), 7.37-7.27 (m, 3H), 4.85 (s, 2H).
^{13}C NMR (100 MHz, CDCl$_3$) δ (ppm) = 168.2, 136.5, 134.1, 132.3, 128.8, 128.7, 127.9 123.4, 41.7.
MS (EI, 70 eV) *m*/*z* (%) = 237.9 (10) [M$^+$], 236.9 (50), 104.0 (100), 91 (27), 77.1 (62).

2-Benzyl-3-oxoisoindoline-1-carbonitrile (6a) [11]

^{1}H NMR (400 MHz, CDCl$_3$) δ (ppm) = 7.84 (d, *J* = 6.7 Hz, 1H), 7.59-7.46 (m, 3H), 7.31-7.14 (m, 5H), 5.40 (d, *J* = 15.1 Hz, 1H), 4.98 (s, 1H), 4.20 (d, *J* = 15.0 Hz, 1H).
^{13}C NMR (100 MHz, CDCl$_3$) δ (ppm) = 167.0, 136.9, 135.3, 133.1, 131.2, 130.5, 129.3, 128.7, 128.5, 124.8, 123.24, 114.6, 48.97, 45.12.
MS (EI, 70 eV) *m*/*z* (%) = 248.9 (20) [M$^+$], 247.9 (100), 144.9 (29), 142.9 (35), 91 (25).

4. References

1 Broggini, G.; Garanti, L.; Molteni, G.; Zecchi, G. A new Entry to [1,2,4]Triazolo[1,5-*a*][1,4]benzodiazepin-6-ones via Intramolecular Nitrilimine Cycloaddition to the Cyano Group. *Tetrahedron* **1998**, *54*, 14859–14868.

2 Majhi, A.; Kim, S.S.; Kadam, S.T. Rhodium (III) iodide hydrate catalyzed three-component coupling reaction: Synthesis of α-aminonitriles from aldehydes, amines, and trimethylsilyl cyanide. *Tetrahedron* **2008**, *64*, 5509–5514.

3 Wang, J.; Masui, Y.; Onaka, M. Synthesis of α-Amino Nitriles from Carbonyl Compounds, Amines, and Trimethylsilyl Cyanide: Comparison between Catalyst-Free Conditions and the Presence of Tin Ion-Exchanged Montmorillonite. *Eur. J. Org. Chem.* **2010**, 1763–1771.

4 Khan, N.H.; Saravanan, S.; Kureshy, R. I.; Abdi, S. H.; Sadhukhan, A.; Bajaj, H. C. Asymmetric addition of trimethylsilylcyanide to *N*-benzylimines catalyzed by recyclable chiral dimeric V(V) salen complex. *J. Organomet. Chem.* **2010**, *695*, 1133–1137.

5 Ramalingam, B.; Seayad, A.M.; Chuanzhao, L.; Garland, M.; Yoshinaga, K.; Wadamoto, M.; Nagata, T.; Chai, C.L.L. A Remarkable Titanium-Catalyzed Asymmetric Strecker Reaction using Hydrogen Cyanide at Room Temperature. *Adv. Synth. Catal.* **2010**, *352*, 2153–2158.

6 Wiles, C.; Watts, P. Evaluation of the heterogeneously catalyzed Strecker reaction conducted under continuous flow. *Eur. J. Org. Chem.* **2008**, 5597–5613.

7 Galletti, P.; Pori, M.; Giacomini, D. Catalyst-free Strecker reaction in water: A simple and efficient protocol using acetone cyanohydrin as cyanide source. *Eur. J. Org. Chem.* **2011**, *2001*, 3896–3903.

8 Jarusiewicz, J.; Choe, Y.; Yoo, K.S.; Park, C.P.; Jung, K.W. Efficient three-component Strecker reaction of aldehydes/ketones *via* NHC-amidate palladium(II) complex catalysis. *J. Org. Chem.* **2009**, *74*, 2873–2876.

9 Ranu, B.C.; Dey, S.S.; Hajra, A. Indium trichloride caralyzed one-step synthesis of—Amino nitriles by a three-component condensation of carbonyl compounds, amines and potassium cyanide. *Tetrahedron* **2002**, *58*, 2529–2532.

10 Nammalwar, B.; Muddala, N.P.; Watts, F.M.; Bunce, R.A. Efficient conversion of acids and esters to amides and transamidation of primary amides using OSU-6. *Tetrahedron* **2015**, *71*, 9101–9111.

11 Dhanasekaran, S.; Suneja, A.; Bisai, V.; Singh, V.K. Approach to isoindolinones, isoquinolinones, and THIQs via Lewis acid-catalyzed domino Strecker-lactamization/alkylations. *Org. Lett.* **2016**, *18*, 634–637.
